# Recovery from Transient Global Amnesia Following Restoration of Hippocampal and Fronto–Cingulate Perfusion

**DOI:** 10.3233/ben-2009-0253

**Published:** 2010-06-29

**Authors:** Paolo Caffarra, Letizia Concari, Simona Gardini, Sabrina Spaggiari, Francesca Dieci, Sandra Copelli, Caterina Ghetti, Annalena Venneri

**Affiliations:** ^1^Department of NeuroscienceUniversity of ParmaParmaItaly; ^2^Outpatient Clinic for the Diagnosis and Therapy of Cognitive DisordersParmaItaly; ^3^Clinical Neuroscience CentreUniversity of HullHullUnited Kingdom; ^4^Servizio di Fisica SanitariaAzienda Ospedaliera di ParmaParmaItaly

**Keywords:** Transient global amnesia, episodic memory, cognitive deficits, SPECT, brain

## Abstract

A patient who suffered a transient global amnesia (TGA) attack underwent regional cerebral blood flow (rCBF) SPECT imaging and neuropsychological testing in the acute phase, after one month and after one year. Neuropsychological testing in the acute phase showed a pattern of anterograde and retrograde amnesia, whereas memory was within age normal limits at follow up. SPECT data were analysed with a within subject comparison and also compared with those of a group of healthy controls. Within subject comparison between the one month follow up and the acute phase detected increases in rCBF in the hippocampus bilaterally; further rCBF increases in the right hippocampus were detected after one year. Compared to controls, significant hypoperfusion was found in the right precentral, cingulate and medial frontal gyri in the acute phase; after one month significant hypoperfusion was detected in the right precentral and cingulate gyri and the left postcentral gyrus; after one year no significant hypoperfusion appeared. The restoration of memory was paralleled by rCBF increases in the hippocampus and fronto-limbic-parietal cortex; after one year neither significant rCBF differences nor cognitive deficits were detectable. In conclusion, these data indicate that TGA had no long lasting cognitive and neural alterations in this patient.

## 1. Introduction

Transient Global Amnesia (TGA) is a neurological condition which is frequently observed in middle age, characterized by sudden onset of amnesia lasting no more than 24 hours [20]. Cognitive assessment during TGA episodes shows a pattern of anterograde and retrograde amnesia with sparing of alertness, attention, personal identity and procedural memory. Several aetiological factors have been suggested such as ischemia, migraine, epileptic phenomena and venous flow abnormalities, although the exact pathogenesis and the aetiology still remain elusive. Uncertainty about the cause of TGA derives from its transient nature and limited duration, features which make the study with complete neuropsychological and neuroradiological work ups in the acute phase challenging. Current clinical criteria prescribe that to make a diagnosis of TGA the attack must be witnessed by an observant, there must be a pattern of clear-cut anterograde amnesia during the at-

ISSN 0953-4180/09,10/$27.50 © 2009/2010 – IOS Press and the authors. All rights reserved tack, clouding of consciousness and loss of personal identity must be absent, cognitive impairment must be limited to amnesia, there should be no accompanying focal neurological symptoms, epileptic seizures must be absent, the attack must resolve within 24 hours and patients have to present no history of recent head injury or known active epilepsy [11].

Neuroimaging studies of cases of TGA in the acute phase have detected functional alterations in the medio-temporal areas, especially in the hippocampal circuit [9]. A case of TGA induced by neurofunctional changes in bilateral medio-temporal lobes has been reported and abnormalities were detected both with MRI and SPECT [6]. A follow-up, two months after the attack, revealed that hippocampal damage was still detectable with MRI, but the bilateral temporal hypoperfusion shown with SPECT in the acute phase had resolved. The authors suggested that in their patient the episode of TGA had been triggered by an ischemic deficit in areas supporting memory processes (i.e. the hippocampus) induced by hemodynamic factors. A SPECT study of TGA in the acute phase showed that the attack was associated with hypoperfusion in the right basal ganglia and left temporal lobe [21]. In detail, changes in brain perfusion in frontal and medial temporal structures, thalamic nuclei and striatum appeared to characterise TGA, indicating that temporary dysfunction of circuits involved in memory encoding and retrieval is critical for the observed memory deficits. Similarly, PET studies during TGA showed brain metabolism changes in frontal and subcortical regions (i.e. lentiform and thalamic nuclei) [2]. There is, therefore, converging evidence both from SPECT and PET studies, that TGA is associated with dysfunction in areas supporting storage and retrieval of episodic memories. MRI data have also confirmed the involvement of memory-related medial temporal structures in TGA [8]. However, to our knowledge there is no reported evidence of dysfunction in other memory-related areas, such as the posterior cingulate cortex, which is also involved in degenerative memory disorders, except for a single case report of TGA due to hemorrhage in the cingulate cortex [23].

The purpose of the present study was to investigate the cognitive and neuro-functional correlates of TGA in the acute phase and at subsequent follow-ups after one month and one year, to investigate whether there were any long term sequels at the neuro-functional level following the attack. To this end, brain perfusion was measured with SPECT and neuropsychological assessments were carried out in a patient who met clinical diagnostic criteria for TGA and presented with a typical pattern, during the acute phase, after one month and after one year from the acute episode.

## 2. Case report

A fifty-five year-old right-handed female teacher with thirteen years of education, was admitted to Parma Maggiore Hospital (Italy) earlier in 2008 with what appeared to be severe amnesia and headache. In her past medical history, she had suffered from rare episodes of cephalalgia, rheumatic fibromyalgia and anxious-depressive episodes treated irregularly with antidepressants (ademethionine). On 12th April 2008 (approximately at 8 a.m.), while at home alone, the patient phoned her daughter but did not recognise her voice and kept asking repetitively for what number she had dialled. Worried by her mother's unusual behaviour, the daughter contacted her sister who at approximately 9 a.m reached her mother's house where she found her a bit disoriented in space and time. She was repetitive, asking the same questions over and over ("What happened?" "Why are you here?" "Why am I not at school?" "My head is bursting", etc.). On the way to the hospital her headache worsened. On examination she did not appear anxious but she claimed that she had been 'dreaming'. She was unable to remember her date of birth, the name of the current prime minister and that recently she had voted during the Italian general political elections (which at that time had just taken place). She had no history of other episodes of this type.

When transferred to the neurological ward (at about 1.30 p.m.), the patient was still clearly amnesic, unable to retain new information. Informal testing of her retrograde memory indicated a severe deficit for recollection of remote events dating back a few weeks. She was disoriented in space and time, and kept on asking the same questions over and over, unable to remember any answers she had been given. She had difficulties in the consolidation and retrieval of new episodic memories, while short-term and semantic memory abilities were normal. General and neurological examinations were normal and her headache had significantly improved compared to few hours earlier.

On the same day she had a brain CT scan, EEG, ECG, routine blood tests, which all showed normal results. The brain perfusion of the patient was also assessed with SPECT on the same day. A qualitative report of this scan revealed a pattern of hypoperfusion in the bilateral inferior temporal regions and right superior and posterior parietal areas. A formal neuropsychological assessment session had been scheduled for 6.30 p.m. after all other assessments mentioned above had taken place, but the patient refused to be involved in any further examination. The pattern which, however, had been revealed by informal assessment, was that of a typical TGA attack, with severe anterograde amnesia, limited retrograde amnesia and sparing of attention, language, praxis and semantic memory.

The following day, the patient appeared oriented in time and space, she was very cooperative and still complained of mild headache. She had no recollection of the events of the day before and it was clear that there was severe retrograde amnesia for the attack. Since symptoms had resolved, twenty four hours after admission she was discharged in good physical conditions. No other remarkable aspects emerged in her personal history, except for her mother having suffered from possible Alzheimer's disease. Follow-up appointments were planned after one month and after one year; on both these sessions she was tested with a comprehensive neuropsychological assessment and brain perfusion scanning with SPECT was repeated.

## 3. Materials and methods

### 3.1. Participants

The patient (female, fifty-five year-old, thirteen years of education) and six healthy individuals (three males and three females, mean age 64.16, SD 8.30; mean education 10.33, SD 6.02) who acted as the control sample for the brain perfusion SPECT analysis took part in this study.

### 3.2. SPECT acquisition and reconstruction

The patient and participants in the control group were injected with 740 MBq of $^{99m}$Tc-HMPAO. One hour after the injection they were imaged with a dual head gamma camera (Adac Genesys Vertex) equipped with FBLF (Fan Beam Long Focus) collimators, with a rotation radius of 15–17 cm. The acquisition matrix was $128 \times 128 \times 16$ with a voxel size of $4.6 \times 4.6 \times 4.6$ mm, the number of projections were 64, with an acquisition time per projection of 40 seconds.

The raw data were reconstructed using a Filter Back Projection technique with a Butterworth 5 order filter and a cut-off frequency of 0.4 cycles cm-1. After reconstruction the images were corrected for attenuation with a Chang first order technique and a linear attenuation coefficient of 0.11 cm-1. To remove any background signal the brains were masked from the images using a 3D ellipsoid-shaped region of interest (ROI). To eliminate low intensity background noise and brain edge image artifacts caused by any partial volume effects, images were cut-off twice below the threshold of 30% of the maximum voxel values and then 70% of the mean voxel value.

These images were saved in DICOM format and then individually normalised to the cerebellum counts using the ImageJ 1.29x software package (National Institutes of Health, USA). This procedure was followed to ensure that the counts in the cerebellum of each individual were the same prior to any processing, which should in turn result in increased sensitivity and specificity of the analysis [19]. The software ImageJ was also used to convert the images into ANALYZE format for subsequent analysis.

### 3.3. Image analysis

Reconstructed images were analysed using the software Statistical Parametric Mapping 5 (SPM5) (Wellcome Trust Centre for Neuroimaging, London, UK). Images were normalised into standard stereotactic space. A 12 parameter affine transformation was used when normalizing images to the standard SPECT template provided in SPM5. Normalised images were then smoothed with an isotropic Gaussian kernel set at 10 mm of full width at half maximum (FWHM).

Three within subject comparisons were carried out with a voxel by voxel subtraction procedure. This analysis allows the observation of within subject modifications over time. The images were normalized into standard stereotactic space and then subtractions were calculated between the one-month follow-up scan and the acute scan, the one year follow-up scan and the acute scan, and the one year follow-up scan and the one month follow-up scan using the ImCulc function of SPM5. This methodology allows the detection of changes in rCBF in the patient from the acute phase after one month and one year.

Further analyses were computed to compare the SPECT images of the patient acquired at different times with those of a group of healthy controls. Differences in regional cerebral blood flow between the patient and the control sample were assessed on a voxel-by-voxel basis using independent sample t-test with age and education included as confounding variables. Three comparisons were carried out, i.e. controls vs acute scan, controls vs one month follow-up scan and controls vs one year follow-up scan.

Table 1
Patient's scores on the neuropsychological tests during the acute phase, after one month and after one year

| Tests | Acute phase | One month after TGA | One year after TGA |
|---|---|---|---|
| MMSE | 18.9 | 27.99 | 28 |
| ADL | 6/6 | 6/6 | 6/6 |
| IADL | 7/8 | 8/8 | 8/8 |
| MDB | | | |
| Progressive Matrices (PM 47) | N.A. | 32.3 | 34.3 |
| Verbal Fluency | N.A. | 26.8 | 26 |
| Verbal Learning Test (15 Rey words) | N.A. | | |
| Immediate | | 34 | 49 |
| Delayed | | 8.5 | 13.5 |
| Visuo-spatial Memory (Rey Figure) | | | |
| Immediate | 20.5 | 34.75 | 33.75 |
| Delayed | 8.50 | 13.75 | 16.25 |
| Semantic Fluency for three categories | 20 | 35 | 43 |
| Short-story recall | | | |
| Immediate | 1.5 | 6.05 | 6.10 |
| Delayed | 0.5 | 5.80 | 4.8 |
| Attentive matrix | N.A. | 44 | 43 |
| Stroop's task | | | |
| Time | N.A. | 16.75 | 17.75 |
| Errors | | 1 | 1 |
| WCST | N.A. | | |
| Category | | 5 | 7 |
| Perseveration errors | 1 | 0 | |
| HAD | | | |
| Anxiety | 13 | 15 | 13 |
| Depression | 10 | 12 | 11 |

N.A.: not administered.

## 4. Results

### 4.1. Cognitive results

#### 4.1.1. Acute phase

As mentioned above, the patient continued to repeat the same sentences over and over and showed temporal disorientation. Anterograde and retrograde episodic memory were impaired; she was unable to store new information and to recall previously learned material. Short-term memory and semantic memory (informally tested) appeared normal (see Table 1).

#### 4.1.2. One month follow-up

After one month, the patient underwent formal neuropsychological testing. Her performance on all tests was within the normal range for her age and education (see Table 1).

#### 4.1.3. One year follow-up

After one year, cognitive testing showed scores remaining in the normal range (see Table 1).

### 4.2. SPECT results

#### 4.2.1. Within-subject analyses

One month after the attack there was increased brain perfusion in the hippocampus, medial temporal lobe and frontal cortex bilaterally (see Fig. 1) when compared with the scan acquired in the acute phase. After one year, there was greater perfusion in the right hippocampus when this scan was compared with the perfusion pattern of the acute scan (see Fig. 2). Higher perfusion in the right hippocampus and frontal cortex was also found when the one year follow-up scan was compared with the one month follow-up scan (see Fig. 3).

#### 4.2.2. Between subject analyses

Acute phase – TGA patient vs controls comparison: Areas of significant hypoperfusion in the patient were detected in the right hemisphere in the precentral (BA 4), cingulate (BA 31) and medial frontal gyri (BA 6) (see Table 2 and Fig. 4).

One month follow-up – TGA patient vs controls comparison: Areas of significant hypoperfusion still persisted in the right precentral (BA 4) and postcentral (BA 5) gyri (see Table 2 and Fig. 5).

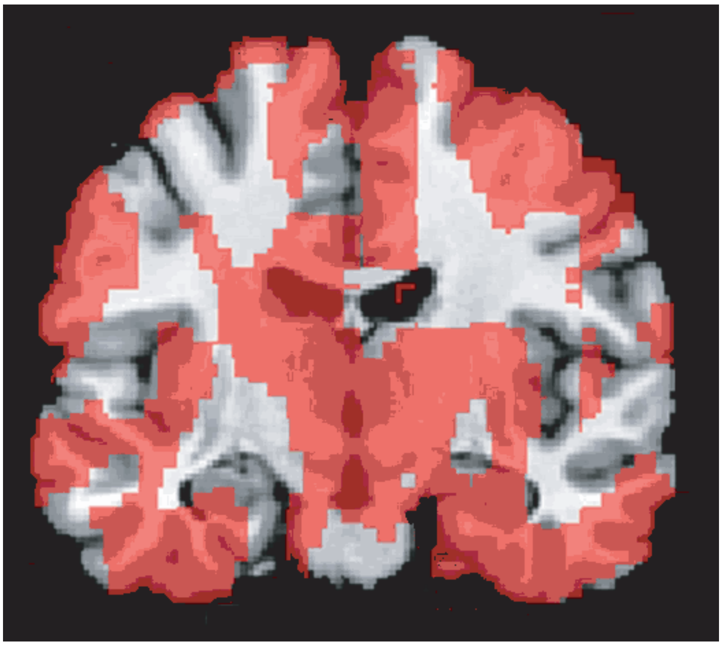

Fig. 1. Areas of differences in perfusion between the patient's scan acquired in the acute phase and that acquired one month after the TGA episode (within subject analysis).

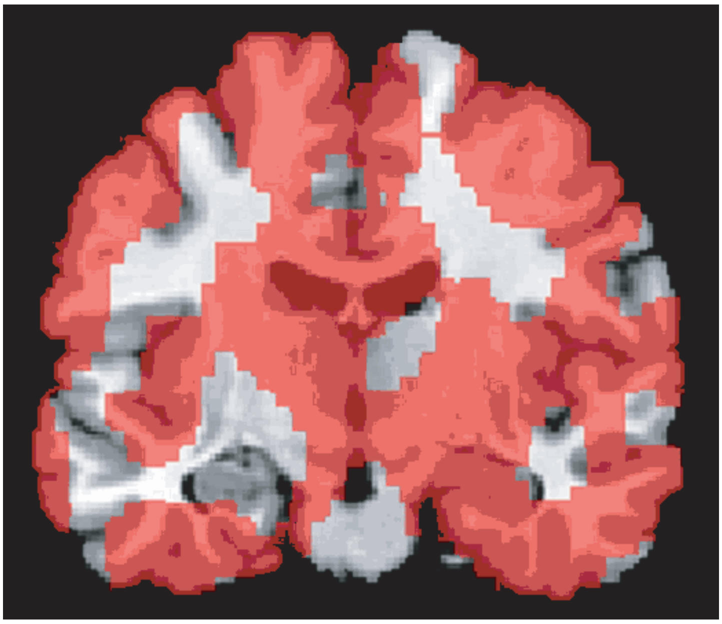

Fig. 2. Areas of differences in perfusion between the patient's scan acquired in the acute phase and that acquired one year after the TGA episode (within subject analysis).

Table 2

Areas of significant hypoperfusion in the TGA patient compared with the control participants in the acute phase, at one month follow-up and at one year follow-up

| Brain Area – Brodman Area (BA) | Number of voxels in cluster | Cluster-level p-value (corrected) | Z value at local maximum | Talairach coordinates x | y | z |
|---|---|---|---|---|---|---|
| **Acute phase** ($p < \mathbf{0.01}$) | | | | | | |
| Right precentral gyrus (BA 4) | 2161 | 0.000 | 4.43 | 30 | −30 | 53 |
| Right cingulate gyrus (BA 31) | | | 4.15 | 10 | −28 | 36 |
| Right medial frontal gyrus (BA 6) | | | 4.01 | 11 | −9 | 52 |
| **One month follow-up** ($p < \mathbf{0.01}$) | | | | | | |
| Right precentral gyrus (BA 4) | 1181 | 0.000 | 4.11 | 30 | −30 | 53 |
| | | | 3.41 | 26 | −20 | 56 |
| Right postcentral gyrus (BA 5) | | | 3.48 | 34 | −44 | 67 |
| **One year follow-up** ($p < \mathbf{0.01}$) | | | | | | |
| No areas of hypoperfusion | | | | | | |

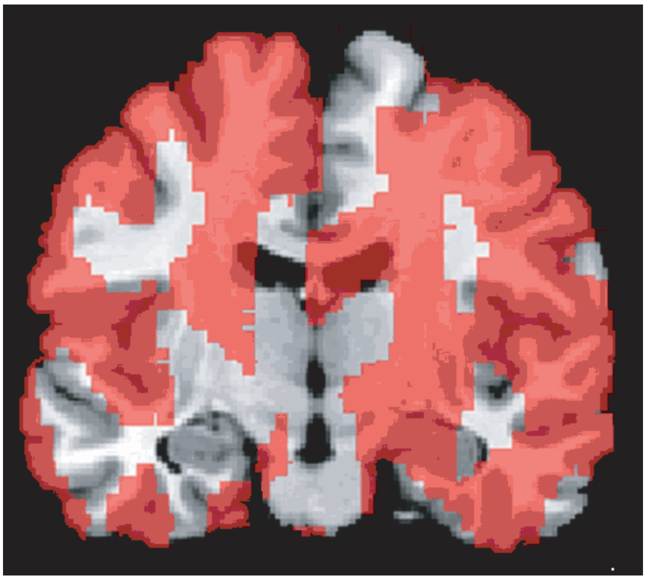

Fig. 3. Areas of differences in perfusion between the patient's scan acquired one month after the TGA episode and that acquired after one year (within subject analysis).

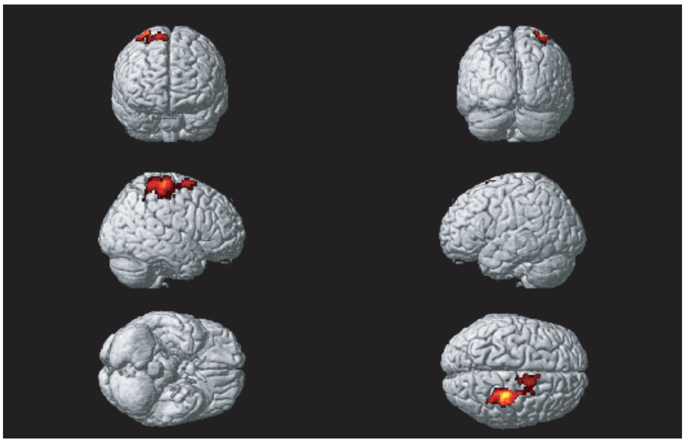

Fig. 4. Areas of significantly lower perfusion in the patient than in controls in the acute phase.

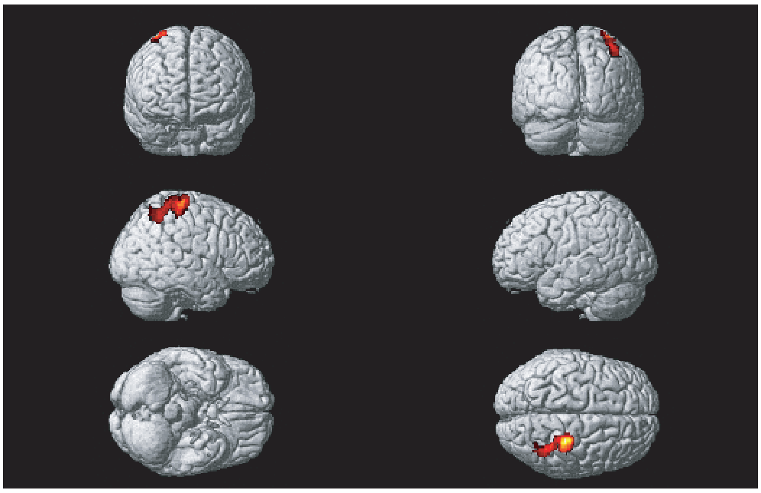

Fig. 5. Areas of significantly lower perfusion in the patient than in controls one month after the TGA episode.

One year follow-up – TGA patient vs controls comparison: There were no areas of significant perfusion difference between the patient and the control sample.

## 5. Conclusion

In the acute phase, the TGA episode of the patient was characterised by severe learning and remote memory retrieval deficits paralleled by significant perfusion reduction in the right precentral, medial frontal and cingulate gyri when compared to healthy matched controls. At follow-up after one month, no abnormalities were observed in her cognitive profile, but a region of significant hypoperfusion confined to the right precentral and postcentral gyri, still persisted. At the subsequent follow-up after one year, no significant neuropsychological or perfusion abnormalities were detectable and the patient appeared fully recovered with no observable cognitive or neuro-functional sequels. Significant increases in activation in area BA 6 in the frontal lobe have been repeatedly reported in fMRI studies of working memory [1,16] and more recently involvement of this area in updating verbal and spatial information has also been suggested [22].

Perfusion deficits in the hippocampus were subtle and not detectable when her scans were compared to those of the healthy controls. Perfusion deficits in this structure as well as other neocortical structures were revealed by the within subject analyses, in particular in the subtraction between the acute scan and that acquired after one month. The increase in perfusion in the hippocampus, in other areas of the medio-temporal cortex and in frontal cortex bilaterally might indicate that the temporary severe amnesia experienced by our patient (in all similar to the typical description of TGA found in clinical guidelines, Caffarra et al. [4]; Hodges and Warlow [11]) was most likely the clinical manifestation of transient dysfunction in medio-temporal and right frontal regions. These increases in brain perfusion, together with those in some regions of the right frontal lobe, might have been of sufficient level to support normal cognitive performance in the patient.

These findings support previous suggestions that transient right frontal dysfunction might subserve episodic retrieval deficits in the acute phase of TGA [2], while temporary diminished function in the hippocampus might be responsible for the profound inability to form new memories. This latter finding would also be consistent with previous reports of neuro-functional changes in medial temporal regions in TGA [6,9,15].

As in most published cases of TGA, also in our patient cognitive performance and brain perfusion returned to normal levels after the episode had resolved [7,13,15], with no detectable lasting residual cognitive or brain perfusion consequences.

The studies which have investigated that TGA episodes might have long term effects have shown controversial findings. In line with the results of this study, some authors reported a positive correlation between the resolution of brain perfusion abnormalities and restoration of normal memory function [12]; others found a dissociation between either the normalization of brain perfusion but persistence of episodic memory impairment [10] or normalization of memory function but persistence of structural brain abnormalities, especially in medial temporal regions [17].

Lampl and colleagues (2004) suggested that the long term effects of TGA might depend on the number of TGA attacks a patients might have had [14]. These authors found that, in the acute phase, TGA patients had areas of hypoperfusion in temporal and thalamic regions. At follow-up brain perfusion returned to normal in those patients who suffered from a single TGA episode, whereas abnormalities remained in those patients who had experienced multiple attacks. Based on this evidence, authors suggested that brain perfusion might be expected to return to normal after a single TGA episode, while normalisation might be less likely when patients experience multiple attacks.

In conclusion, the findings from this case study suggest that a single TGA episode has no long lasting cognitive or neurodynamic consequences and full cognitive and functional recovery is possible. TGA, therefore, appears to be a benign syndrome resulting from transient dysfunction of structures supporting memory encoding and retrieval processes such as the hippocampus (bilaterally), the right prefrontal and cingulate cortex. However in a few cases the differential diagnosis of TGA should include also an early stage of cognitive impairment evolving to Alzheimer's disease [3,5].

This study was not able to attribute a precise aetiology to the TGA. In this patient, the amnestic attack might have been related to a circulatory disorder associated with cephalalgia or to emotional factors (in line with Quinette et al. [18]). Further longitudinal studies are necessary to clarify the aetiology of TGA, the long term effects on memory and its possible relationship with the occurrence of other pathological conditions, such as dementia.

## Acknowledgments

The authors thank the patient for participating in the longitudinal clinical and neuroimaging assessments and the Neuroradiology division of the Hospital of Parma for their assistance with the imaging study. This study was supported by FIL (ex 60%) funding and by a grant from Fondazione Cassa di Risparmio of Parma to PC.
